# Opposing Synovial Cannabinoid Receptor Type 2 and Transient Receptor Potential Vanilloid 1 Expression in Painful Osteoarthritis

**DOI:** 10.7759/cureus.92144

**Published:** 2025-09-12

**Authors:** Collin A Toups, Lauren G Guillot, Kaitlyn Redondo, Sydney Jensen, Jennifer Klein, Luis Marrero

**Affiliations:** 1 School of Medicine, Louisiana State University Health Sciences Center, New Orleans, USA; 2 Department of Orthopedic Surgery, Louisiana State University Health Sciences Center, New Orleans, USA; 3 Department of Biochemistry, Louisiana State University Health Sciences Center, New Orleans, USA

**Keywords:** cannabinoid receptor 2, chronic joint pain, chronic synovitis, knee osteoarthritis (koa), transient receptor potential vanilloid 1

## Abstract

Knee osteoarthritis (kOA) poses a major health challenge worldwide, but current non-surgical treatments have limited long-term success and do not address the underlying disease process. This study explores the complex relationship between the endocannabinoid system (ECS) and the endovanilloid system (EVS) in the synovial membrane of patients with kOA undergoing total knee replacement, focusing on how these systems relate to patient-reported pain and histological synovitis. Synovial tissues and fluid from patients grouped into high and low pain categories based on the Knee Injury and Osteoarthritis Outcome Score (KOOS) were examined for cannabinoid receptor type 2 (CB2R), transient receptor potential vanilloid 1 (TRPV1), protein gene product 9.5 (PGP9.5), calcitonin gene-related peptide (CGRP), and endocannabinoids (2-arachidonoylglycerol (2-AG) and anandamide (AEA)). Results show that higher reported pain and more severe synovitis are linked to significantly lower levels of synovial CB2R and higher TRPV1 expression. Higher levels of synovial fluid (SF) 2-AG were also found in high-pain groups, along with a trend toward increased CGRP levels. The distribution of PGP9.5 did not differ significantly between groups. These findings collectively suggest a dysregulated TRPV1/CB2R axis in painful kOA synovitis. This research offers key descriptive data and important initial insights into the molecular landscape of painful kOA, indicating potential for targeted CB2R-specific treatments to help manage pain and inflammation.

## Introduction

Knee osteoarthritis (kOA) stands as a leading cause of chronic disability worldwide, impacting over 595 million individuals globally in 2020 [1] and incurring an estimated annual socioeconomic cost exceeding $20 billion in the United States alone [2]. Projections indicate a substantial increase in total knee arthroplasty (TKA) procedures, potentially surpassing 3.4 million annually by 2040 in the United States [3], highlighting the complexities and limitations of current postoperative pain management strategies in TKA [4] and underscoring the urgent need for more effective non-operative therapeutic interventions to improve clinical outcomes and potentially delay or prevent the need for surgical intervention.

Current non-operative management strategies for kOA primarily focus on symptomatic relief through the use of anti-inflammatory and analgesic agents, such as non-steroidal anti-inflammatory drugs (NSAIDs) and intra-articular corticosteroids [5]. While these treatments can offer temporary alleviation of symptoms, they frequently lack long-term efficacy and do not fundamentally alter the progressive course of the disease. Furthermore, concerns exist regarding their potential to accelerate disease progression, for instance, through chondrotoxic effects or by masking pain, which may lead to joint overuse [6]. Chronic use of these agents also carries risks of systemic side effects [7]. Critically, no currently available non-operative therapies effectively attenuate the progressive tissue destruction, persistent low-grade inflammation, and detrimental fibrosis that characterize the pathogenesis of kOA. This significant unmet clinical need necessitates the development of disease-modifying therapeutics for kOA that can provide durable pain relief while simultaneously halting or slowing disease progression [8].

Pain and stiffness, the hallmark symptoms of kOA, exhibit considerable heterogeneity among patients and often correlate poorly with the degree of structural disease progression assessed via clinical or radiological examination [9]. This discrepancy suggests that damage to radiopaque structures, such as cartilage and bone, alone does not fully account for the patient's symptomatology [10]. One quantifiable factor influencing pain perception is the presence and activity of nociceptive fibers within the inflamed synovial membrane. An increased density of these fibers, often identified by protein gene product 9.5 (PGP9.5) expression, is strongly associated with painful kOA pathogenesis [11].

The transient receptor potential vanilloid 1 (TRPV1) channel, a non-selective cation channel and a component of the endovanilloid system (EVS) (pain signaling sensor and modulator network), is expressed on these nociceptive fibers and non-neuronal cells, such as fibroblast-like synoviocytes [12]. TRPV1 activation occurs in response to various noxious stimuli, including heat, inflammatory cytokines (e.g., tumor necrosis factor-alpha (TNF-α) and interleukin-1 beta (IL-1β)), lipid mediators, capsaicin, and the acidic pH characteristic of acute and chronic inflammatory states [13]. This activation leads to the depolarization of afferent nociceptors, triggering the release of pain signals, reactive oxygen species (ROS), and neuropeptides such as calcitonin gene-related peptide (CGRP) and substance P, which collectively promote neurogenic inflammation and hyperexcitability [14].

Therapeutic strategies directly targeting TRPV1 have yielded mixed results. TRPV1 agonists, such as topical capsaicin, provide temporary analgesia by activating and desensitizing the nociceptive pathway through receptor internalization and defunctionalization [15]. However, this relief is often accompanied by intense burning sensations, which can limit patient compliance [16]. Selective TRPV1 antagonists have demonstrated analgesic efficacy in animal models for inflammatory pain conditions. However, human trials in kOA were halted due to adverse effects, including impaired heat sensation and hyperthermia, which is likely due to TRPV1's integral role in regulating core body temperature [17]. The limitations of directly targeting TRPV1 underscore the need for non-opioid alternative approaches to modulate the EVS for managing multifactorial kOA pain.

The endocannabinoid system (ECS), a widespread neuromodulatory network comprising cannabinoid receptors (CB1R and CB2R), their endogenous ligands (endocannabinoids such as anandamide (AEA) and 2-arachidonoylglycerol (2-AG)), and metabolic enzymes, interacts extensively with the EVS, offering an alternative regulatory pathway [18]. Its role in pain, inflammation, and immune regulation, particularly in rheumatic diseases, positions it as a significant therapeutic target [19]. Both CB1R and CB2R modulate pain and inflammation, partly through interactions with TRPV1, with direct evidence of CB2R and TRPV1 co-localization and functional interaction in human sensory neurons [20]. AEA, a well-known endocannabinoid and endogenous agonist of CB1R and CB2R, also binds to TRPV1 with an affinity similar to capsaicin, effectively acting as an "endovanilloid" [21], with detailed molecular insights into its binding modes [22]. Co-activation of CB2R and TRPV1 by AEA, or through other indirect mechanisms, can result in a smaller calcium influx through TRPV1 than selective activation of TRPV1. This attenuated signal appears sufficient to induce receptor dysregulation and desensitization, producing an analgesic effect without the intense burning associated with direct, potent TRPV1 agonists, such as capsaicin. This desensitization can also occur via TRPA1 activation [23]. Interestingly, levels of 2-arachidonoylglycerol (2-AG), another well-known endocannabinoid and endogenous agonist of CB1R and CB2R, are increased in the setting of TRPV1 activation [24]. While exogenous cannabidiol (CBD), AEA, and 2-AG can modulate CB2R and TRPV1 dynamics, they may also activate the psychotropic CB1R, predominantly expressed in the central nervous system but also present peripherally, leading to psychoactive alterations. CBD, specifically, can act as a negative allosteric modulator of the CB1 receptor, which can reduce the efficacy and potency of CB1R agonists and potentially mitigate psychoactive effects [25]. Furthermore, long-term systemic CBD administration carries potential hepatotoxicity risks [26]. Despite these concerns, CBD has emerged as a promising therapeutic agent due to its diverse pharmacological properties, including potent anti-inflammatory, neuroprotective, and immunomodulatory effects [27].

However, novel, high-affinity CB2R-specific agonists present a promising targeted alternative. These agents primarily target CB2R, which is abundantly expressed on immune cells and fibroblasts, and, minimally, in peripheral nerve terminals and other tissues [23,28]. Such agents could leverage the TRPV1 crosstalk mechanism to achieve anti-inflammatory, anti-fibrotic, and analgesic effects in kOA without the psychoactive, off-target, or hepatotoxic liabilities associated with CB1R activation or systemic CBD application.

kOA pain is frequently linked to synovitis. Synovial inflammation promotes pain sensitization through the local release of inflammatory mediators, including cytokines, chemokines, prostaglandins, and growth factors, leading to neuronal hypersensitivity and increased nociceptive inputs from the joint [29,30]. This necessitates investigation of receptor dynamics in this inflamed environment. The pro-nociceptive role of TRPV1 is established; conversely, the ECS, particularly via CB2R, offers counter-regulation. That is, by modulating pro-inflammatory signaling (e.g., by inhibiting cytokine release from synovial macrophages and fibroblasts), activated CB2R can indirectly attenuate the conditions (e.g., an inflammatory microenvironment and acidic pH) that are conducive to TRPV1 activation. However, the interplay of these systems in kOA synovium, particularly in relation to patient-specific pain and synovitis severity, requires detailed investigation. This study was designed to elucidate these relationships and identify variations in ECS and EVS components related to kOA pain, to determine potential patient responsiveness to therapeutic CB2R-targeted analog supplementation for painful synovitis. We predicted that (1) higher patient-reported pain on the Knee injury and Osteoarthritis Outcome Score (KOOS) pain subscale and more severe synovitis would correlate with increased synovial TRPV1 expression, reflecting heightened nociceptive signaling in an inflammatory state; (2) as TRPV1 activation elevates 2-AG, increased synovial fluid (SF) 2-AG was predicted in high-pain cohorts [24]; and (3) for CB2R, despite its protective functions, it was predicted that chronic synovitis and sustained 2-AG production and local accumulation, via established principles of agonist-induced receptor downregulation and desensitization [31], paradoxically reduce CB2R expression, thereby impairing its protective pathway. To test these hypotheses, banked synovial tissues collected from patients with kOA undergoing TKA were evaluated for TRPV1, PGP9.5, and CB2R distribution relative to KOOS pain and synovitis scores, and SF was assayed for 2-AG. The goal is to guide patient responsiveness to targeted, non-surgical, CB2R-specific treatments relative to pain sensation attributable to kOA-related synovitis.

## Materials and methods

Patients

This study, utilizing prospectively collected samples and clinical data, received approval from the Institutional Review Board (IRB) at Louisiana State University Health Sciences Center-New Orleans (LSUHSC-NO) (IRB #986). All participants provided written informed consent before their inclusion. The LSU Integrated Musculoskeletal Biobank (LIMB) systematically collects knee tissues, synovial fluid (SF), and blood from patients with end-stage (Kellgren-Lawrence score of 4) kOA undergoing TKA. Stored biospecimens and relevant histological metrics are integrated with patient records, functional measures, and patient-reported outcome questionnaires. Eligible patients underwent primary TKA for kOA, confirmed by clinical and radiographic criteria, with all procedures performed by a single, fellowship-trained surgeon using standardized techniques to minimize variability. Preoperative parameters collected included race, age, sex, body mass index (BMI), comorbidities, range of motion, and deformity.

Reported pain measures

KOOS subscales for symptoms, pain, activities of daily life, and knee-related quality of life were collected approximately four weeks before surgery. This study specifically utilized the pain subscale, scored from 0 (extreme knee pain) to 100 (no knee pain). Patients were categorized based on pain severity using quartiles calculated from the entire LIMB biobank KOOS pain score distribution. This study focused on comparing samples from patients (N = 73) in the highest pain quartile (Q1 ≤ 22.20, representing high pain, n = 41) and the lowest pain quartile (Q3 ≥ 47.20, representing low pain, n = 32) from a banked set of 350 patients with kOA. It is recognized that all patients in this cohort underwent TKA, implying that they all experienced pain severe enough to warrant surgical intervention. However, within this population of patients with end-stage kOA, there remains a broad spectrum of individual pain perception and severity. The utilization of quartiles from a larger biobank of 350 patients allowed for a robust stratification of "high" versus "low" pain within the context of end-stage OA requiring surgery. This stratification revealed distinct biological differences in molecular profiles, suggesting that even within a clinically significant pain range, measurable variations correlate with subjective pain levels. This approach is valuable for identifying potential biomarkers that might predict responsiveness to targeted therapies, even among patients with severe disease, by differentiating underlying molecular profiles associated with differing pain experiences.

Synovitis scoring

The severity of synovitis was staged histologically from paraffin-embedded sections of suprapatellar synovium. Five-micrometer sections of formalin-fixed, paraffin-embedded synovium were stained with the Picrosirius (PS) Red technique, which can be used to score for synovitis [32] similar to hematoxylin and eosin (H&E) staining, based on criteria established by Krenn et al. [33]. This method evaluates three key features: hyperplasia of the synovial lining layer, cellularity of the synovial stroma (resident cell density), and inflammatory infiltrate across 20 transmitted microscopy fields at 200× magnification, including intimal and subintimal layers as previously shown [32,33]. A composite score (ranging from 0 to 9) was generated, with zero indicating absence of synovitis and 9 indicating severe synovitis. Scoring was performed independently by two blinded observers, and inter-rater reliability was assessed using the weighted Cohen's kappa coefficient (κ). For analysis, scores were categorized into low-grade (0-4) and high-grade (5-9) groups [32].

Quantitative immunofluorescence (Q-IF)

Quantitative immunofluorescence (Q-IF) was performed on two sets of paraffin-embedded synovial tissue sections adjacent to those stained by PS, corresponding to each patient in cohorts grouped by KOOS pain quartiles. After deparaffinization and rehydration, epitope retrieval was performed by submerging slides in a pH 5.1-5.5 citrate buffer and heating. Sections were blocked with 5% goat serum in phosphate-buffered saline (PBS) to prevent non-specific antibody binding and incubated overnight at 4°C with primary antibodies. One set of slides was incubated with rabbit anti-CB2R (Abcam, Cambridge, UK; polyclonal; ab3561). The second set was co-incubated with rabbit anti-TRPV1 (Abcam; polyclonal; ab3487) and mouse anti-PGP9.5 (Abcam; monoclonal (13C4/I3C4); ab8189). For co-labeled slides, secondary antibodies were Alexa Fluor 647-conjugated anti-rabbit IgG (for TRPV1) and Alexa Fluor 594-conjugated anti-mouse IgG (for PGP9.5). CB2R sections were detected using Alexa Fluor 594-conjugated goat anti-rabbit IgG. Nuclei were counterstained with 4’,6-diamidino-2-phenylindole (DAPI), and slides were mounted. Isotype-matched negative control antibodies (rabbit IgG and mouse IgG) were used at equivalent concentrations for all primary antibodies to assess non-specific binding and autofluorescence. Antibody validation was performed through careful optimization of concentrations and washing steps to minimize non-specific binding, building on the manufacturer's validation and previous literature. Images were acquired from two representative fields per sample using an FV1000 laser scanning confocal microscope at 200× magnification, utilizing 405 nm, 592 nm, and 633 nm laser lines and corresponding photodetectors. Background-corrected target signal intensity was quantified using Slidebook® software. Fluorescence signals for CB2R, TRPV1, and PGP9.5 were normalized to cell number (determined by DAPI-stained nuclei count in the same field) to account for variations in cellularity over fields with the same area. While TRPV1 expression was assessed in relation to PGP9.5+ nerve fibers and noted on non-neuronal cells, further image analysis is underway to quantify TRPV1 specifically within neuronal and non-neuronal compartments for more precise insights into cellular localization. Similarly, co-staining CB2R with PGP9.5 is being explored to determine its expression by nerves in the synovium. These detailed quantifications and co-staining analyses are part of ongoing studies to identify specific CB2R-positive cell populations within the synovium.

Enzyme-linked immunosorbent assay (ELISA)

Synovial fluid (SF) samples were first treated with hyaluronidase to reduce viscosity and then centrifuged to remove cellular debris, and the supernatant was collected. Samples were assayed neat and in triplicate for calcitonin gene-related peptide (CGRP) using a sandwich ELISA kit (Cusabio Biotech Co., Ltd.; Wuhan, China; E08210h) according to the manufacturer's protocol. Briefly, standards and samples were incubated on the antibody-pre-coated plate, followed by washing and incubation with a biotinylated detection antibody. After washing, avidin-horseradish peroxidase (HRP) conjugate was added. Following final washes, a tetramethylbenzidine (TMB) substrate solution was added, and the enzymatic reaction was stopped with dilute sulfuric acid. Absorbance was read at 450 nm using a xMark microplate reader (Bio-Rad, Hercules, CA), and concentrations reported in pg/mL were calculated from optical density values using a four-parameter logistic (4PL) regression model. CGRP was measured in synovial fluid as an indirect marker of peripheral TRPV1 activity, as TRPV1 activation on sensory nerves stimulates the release of CGRP. Tissue staining for CGRP, while providing valuable anatomical localization, was not performed in this study, which focused on soluble mediators in SF.

Liquid chromatography-tandem mass spectrometry (LC-MS/MS) for endocannabinoid quantification

Deuterated 2-arachidonoylglycerol (2-AG-d8) and N-arachidonoylethanolamide (AEA-d4) were used as internal standards for quantification. Stock solutions were prepared in methanol and stored at -80°C. Samples were prepared using MS-grade acetonitrile (ACN), chloroform, and methanol. SF (100 µL) samples were spiked with 5 µL of each deuterated standard stock solution for lipid extraction. The samples were then mixed with ddH2O, methanol, and chloroform (a modified Bligh-Dyer liquid-liquid extraction) and centrifuged to separate the phases. The lower organic phase was transferred, dried using a speed vac, resuspended in 15 µL of 50% methanol, and transferred to a sample vial for analysis. Ultra performance liquid chromatography-tandem mass spectrometry (UPLC-MRM-MS/MS) analysis was performed using a 1290 Infinity II LC system coupled with a 6495 LC/TQ (triple quadrupole) mass spectrometer (Agilent Technologies, Santa Clara, CA). A ZORBAX Eclipse Plus C18 column was used for reverse-phase separation. Solvent A was 0.1% formic acid in ddH2O, and solvent B was 0.1% formic acid in ACN. The mobile phase flow rate was 0.4 mL/minute, and the injection volume was 8 µL. A detailed gradient elution program was followed. Molecules were analyzed using a triple quadrupole mass spectrometer equipped with a Jet Stream electrospray ionization (ESI) source, operating in positive ionization mode. Endocannabinoids were detected in positive mode using a multiple reaction monitoring (MRM) technique to enhance selectivity and sensitivity. Source conditions were optimized. Four MRM transitions were monitored, corresponding to 2-AG, its deuterated internal standard 2-AG-d8, AEA, and its deuterated internal standard AEA-d4. Collision energy was optimized and set to 15 eV for all transitions. Dwell time was 500 ms for 2-AG and 2-AG-d8 and 50 ms for AEA and AEA-d4. MassHunter Quantitative Analysis 10.0 software was used for all LC-MS/MS data acquisition and processing, with concentrations calculated directly from the ratio of peak areas corresponding to the analyte and its respective heavy deuterated internal standard.

Statistics

Statistical analyses were performed using Prism 8 (GraphPad Software). Normally distributed continuous data were analyzed using independent two-sample t-tests and presented as mean ± standard deviation (SD). Non-normally distributed data were analyzed using the Mann-Whitney U test, with results reported as median and interquartile range (IQR). Pearson's correlation coefficient (r) assessed associations between continuous, normally distributed variables. Linear regression analysis was used to examine the relationship between synovitis score and CB2R expression, reporting the coefficient of determination (R2) and F-statistic to assess the model's goodness of fit. Univariable analyses were performed as no significant differences were found in baseline demographics or preoperative clinical measures between pain groups, simplifying the analytical approach. Statistical significance was set at α = 0.05 for all analyses. Bar plots with individual data points were primarily used for clarity in presenting mean ± SD and individual patient variability for key quantitative measures. Violin plots were used where distribution shape and density across the range of values were particularly informative, such as for synovitis scores, to better visualize the spread and density of data points within each quartile, complementing the mean ± SD.

## Results

Patient characteristics

The study cohort (N = 73) comprised 67.1% female (n = 49) and 32.9% male (n = 24) patients undergoing TKA for end-stage kOA. The self-reported race distribution was 63% White (n = 46), 33% Black (n = 24), and 4% other (n = 3). The median age of the cohort was 67.5 years (range: 40-87 years), and the mean body mass index (BMI) was 30.9 kg/m² (range: 23.2-43.6 kg/m²), indicating that the patient population was, on average, overweight to obese. As stated in the methods, no significant differences were observed in these baseline demographics or preoperative clinical measures between the subsequently defined low- and high-pain groups. This matching of cohorts strengthens the conclusion that the observed molecular differences are associated with the pain phenotype itself, rather than a demographic or clinical imbalance. Table 1 provides a comprehensive overview of patient demographics and clinical characteristics by KOOS pain group.

**Table 1:** Patient demographics and clinical characteristics by KOOS pain group KOOS: Knee Injury and Osteoarthritis Outcome Score, BMI: body mass index, SD: standard deviation

Characteristic	Total cohort (N = 73)	Low-pain group (KOOS Q3, n = 32)	High-pain group (KOOS Q1, n = 41)	p-value (high versus low)
Sex (number (%))				
Female	49 (67.1)	22 (68.8)	27 (65.9)	0.79
Male	24 (32.9)	10 (31.3)	14 (34.1)	
Race (number (%))				
White	46 (63.0)	20 (62.5)	26 (63.4)	0.93
Black	24 (32.9)	11 (34.4)	13 (31.7)	
Other	3 (4.1)	1 (3.1)	2 (4.9)	
Median age (years) (range)	67.5 (40-87)	68.0 (40-87)	67.0 (46-85)	0.74
Mean BMI (kg/m²) (range)	30.9 (23.2-43.6)	30.7 (23.2-42.1)	31.1 (24.5-43.6)	0.68
Mean KOOS pain score (± SD)	35.8 (± 14.1)	54.2 (± 7.5)	20.3 (± 3.8)	<0.0001
Mean synovitis score (± SD)	4.7 (± 1.2)	4.2 (± 0.9)	5.2 (± 1.2)	0.0002

Perceived pain and synovitis severity

**Figure 1:**
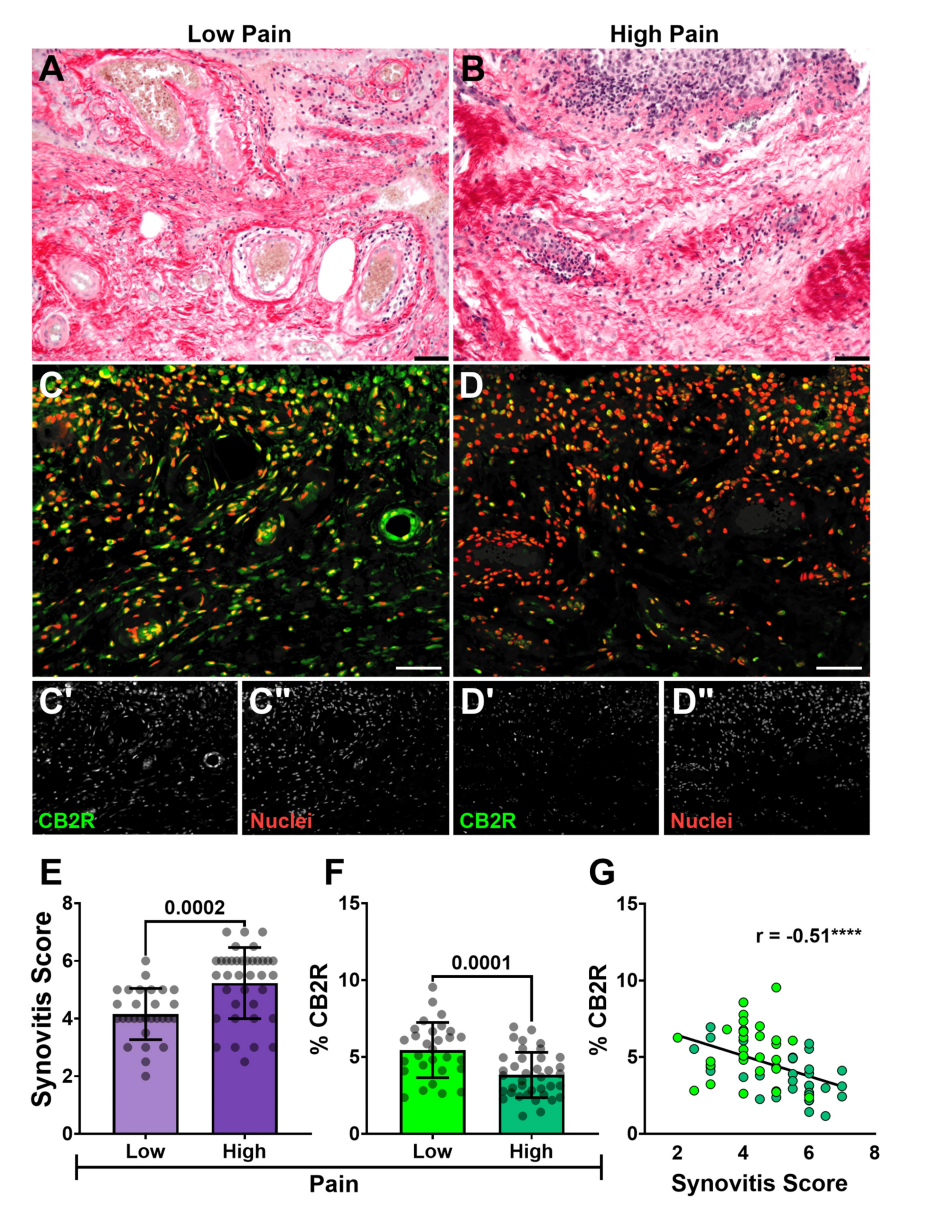
Histological synovitis and CB2R distribution relative to KOOS pain (A) Representative PS-stained sections of synovium from patients showing synovitis severity corresponding to (A) low (KOOS Q3 ≥ 47.20) and (B) high (KOOS Q1 ≤ 22.20) self-reported pain (bars = 50 µm). Confocal photomicrographs captured at 200× corresponding to PS-stained synovium sections of patients from the (C) low and (D) high-pain cohorts stained for CB2R (green) (C’ and D’ in grayscale) and nuclei (red) (C” and D” in grayscale) (bars = 100 µm). (E) Comparison of mean ± SD synovitis score between low and high KOOS pain groups (Student's t-test, α =0.05). (F) Bar graph with individual data points comparing mean ± SD percentage of CB2R signal (normalized to cell number) between KOOS pain groups (Student's t-test, α =0.05). (G) A scatterplot with linear regression analysis demonstrates a negative correlation between synovitis score and histological CB2R expression. Pearson correlation coefficient (r) with ***p < 0.0001. PS: Picrosirius, KOOS: Knee Injury and Osteoarthritis Outcome Score, CB2R: cannabinoid receptor type 2, SD: standard deviation

Histological synovitis scoring, using the Krenn score, performed by two independent blinded observers, yielded high inter-rater agreement (weighted κ = 0.9 and 0.8, indicating excellent and reasonable agreement, respectively) [32]. Patients in the high-pain group (KOOS Q1) exhibited significantly higher mean synovitis scores (5.2 ± 1.2) compared to the low-pain group (KOOS Q3) (4.2 ± 0.9) (p = 0.0002) (Figure 1A, 1B, 1E). This indicates a positive association between the severity of synovial inflammation and patient-reported pain levels.

CB2R localization in the synovium

Synovial CB2R distribution, as measured by Q-IF intensity normalized to cell number and expressed as a percentage of total tissue area, was significantly lower in the high-pain group (3.8 ± 1.5%) compared to the low-pain group (5.4 ± 1.8%) (p = 0.0001) (Figure 1F). Furthermore, the expression of this receptor showed a strong negative correlation with the synovitis score (r(63) = -0.5107, p = 0.0001). This inverse relationship was further confirmed by linear regression analysis (F(1,63) = 15.8, p = 0.0002), with an R2 value of 0.2 indicating that approximately 20% of the observed variability in synovitis severity could be statistically explained by CB2R expression (Figure 1G). Collectively, these data demonstrate that diminished synovial CB2R levels are associated with both higher patient-reported pain and increased histological synovitis.

TRPV1 density relative to sensory nerves and synoviocytes

**Figure 2:**
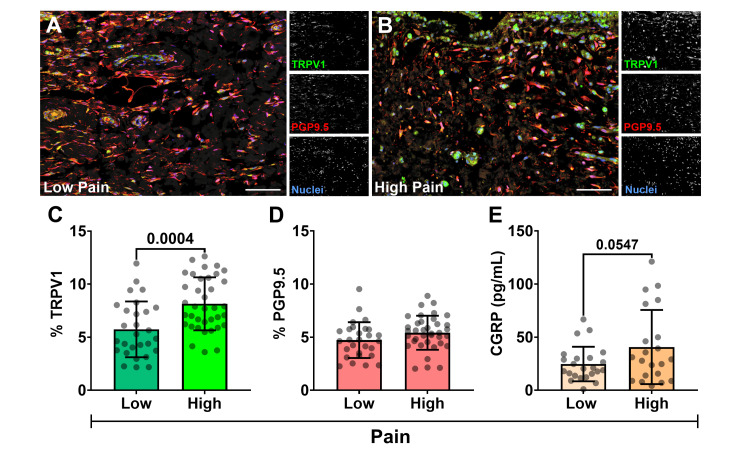
TRPV1 and PGP9.5 neural distribution relative to KOOS pain (A) Representative 200× confocal photomicrographs of co-labeled TRPV1 (green), PGP9.5 (red), and nuclei (blue) in representative patient synovium from the (A) low-pain (KOOS Q3 ≥ 47.20) and (B) high-pain (KOOS Q1 ≤ 22.20) cohorts. Corresponding grayscale channels are shown to the right of A and B (bar = 100 µm). Bar graphs with individual data points comparing mean ± SD expression percentage from Q-IF detection of (C) TRPV1 distribution and (D) PGP9.5 nerve fibers and synoviocytes (normalized to cell count) between KOOS pain groups (Student's t-test, α = 0.05). (E) Bar graph with individual data points comparing CGRP concentration (pg/mL) in SF measured by ELISA, between KOOS pain groups (Student's t-test, α =0.05). TRPV1: transient receptor potential vanilloid 1, PGP9.5: protein gene product 9.5, KOOS: Knee Injury and Osteoarthritis Outcome Score, SD: standard deviation, Q-IF: quantitative immunofluorescence, CGRP: calcitonin gene-related peptide, SF: synovial fluid, ELISA: enzyme-linked immunosorbent assay

Q-IF analysis revealed TRPV1 expression co-localizing with PGP9.5+ sensory nerve fibers, predominantly in the synovial subintima and within synoviocytes of the synovial lining layer (Figure 2A, 2B). For quantitative assessment, fluorescence signals for TRPV1 and PGP9.5 were normalized to cell number and expressed as a percentage of the total tissue area. This quantitative analysis demonstrated significantly higher mean TRPV1 in the high-pain group (KOOS Q1, lowest KOOS scores; 8.1 ± 2.5%) compared to the low-pain group (KOOS Q3, highest KOOS scores; 5.7 ± 3.0%) (p = 0.0004) (Figure 2C). In contrast, the density of synovial nerve fibers, assessed by PGP9.5 expression, did not significantly differ between the low-pain (4.7 ± 1.7%) and high-pain (5.4 ± 1.6%) groups (p = 0.1) (Figure 2D). These results suggest that the increased TRPV1 expression observed in patients with higher pain is attributable to greater receptor levels on existing nerves and/or synoviocytes, rather than an overall increase in nerve fiber density. This points to a qualitative change in the nociceptive apparatus, characteristic of peripheral sensitization, rather than a simple quantitative increase in innervation.

TRPV1 activation measured by CGRP output

CGRP levels in SF (n = 44 patients; 21 high pain and 23 low pain), measured by ELISA, served as an indirect marker of peripheral TRPV1 activity, as TRPV1 activation on sensory nerves stimulates CGRP release (Figure 2E). A statistically significant, albeit weak, negative linear correlation was observed between SF CGRP concentrations and KOOS pain scores (where lower KOOS scores indicate higher pain) (r(42) = -0.303, p = 0.045). This suggests that higher CGRP levels were associated with higher self-reported pain. When comparing the means between the KOOS pain quartiles, the difference in CGRP levels were nearly significant, with higher mean concentration in the high-pain group (low pain: 24.7 ± 16.2 pg/mL versus high pain: 40.7 ± 23.5 pg/mL; p = 0.0547) (Figure 2E).

2-AG and AEA levels in SF

**Figure 3:**
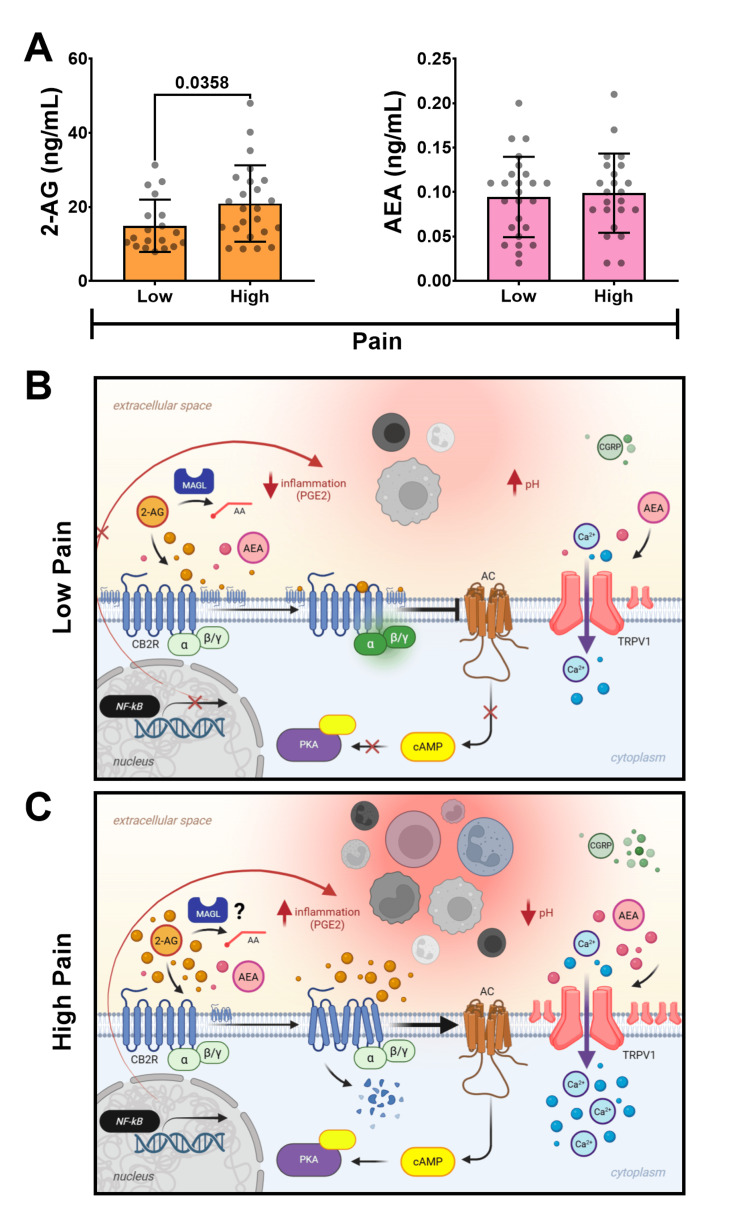
Synovial fluid endocannabinoid levels and hypothesized signaling in low versus high pain states (A) SF concentrations of 2-AG and AEA (ng/mL) measured by LC-MS/MS, compared between low and high KOOS pain groups. 2-AG is significantly elevated in the high-pain group. (B) Hypothesized signaling in a low pain state with low-grade synovitis: normal CB2R expression and physiological 2-AG levels promote CB2R activation via the Gαi/o pathway. This inhibits adenylyl cyclase, reduces cAMP, and suppresses NFκB activation, decreasing pro-inflammatory cytokine production and reducing inflammation/synovitis. TRPV1 activity is likely to be at baseline or appropriately modulated, contributing to lower pain perception. Graphic created in BioRender: Marrero, L. (2025) https://biorender.com/wdd2owj [34]. (C) Hypothesized signaling in a high pain state with high-grade synovitis: increased inflammation and an acidic microenvironment lead to TRPV1 overactivation. This drives supra-physiological 2-AG production and accumulation. Sustained high 2-AG levels lead to chronic CB2R activation, desensitization, downregulation (reduced receptor amounts), and a potential paradoxical shift to pro-inflammatory Gαs signaling. This impairs CB2R's protective effects, potentially increasing NFκB activity and pro-inflammatory cytokine production, exacerbating synovitis, TRPV1 sensitization, and pain. Graphic created in BioRender: Marrero, L. (2025) https://biorender.com/aoj686t [35]. SF: synovial fluid, 2-AG: 2-arachidonoylglycerol, AEA: anandamide, LC-MS/MS: liquid chromatography-tandem mass spectrometry, KOOS: Knee Injury and Osteoarthritis Outcome Score, CB2R: cannabinoid receptor type 2, cAMP: cyclic adenosine monophosphate, NFκB: nuclear factor kappa-light-chain-enhancer of activated B cells, TRPV1: transient receptor potential vanilloid 1

LC-MS/MS analysis of endocannabinoids in SF revealed significantly higher levels of 2-AG in patients in the high-pain group (20.9 ± 10.3 ng/mL) compared to the low-pain group (14.9 ± 7.1 ng/mL) (p = 0.0358) (Figure 3A). Conversely, similar concentrations of AEA were observed between high (0.99 ± 0.05 ng/mL) and low (0.94 ± 0.04 ng/mL) pain groups (p > 0.05) (Figure 3A).

## Discussion

Driven by the pressing clinical need for more effective and durable treatments for kOA, this study provides compelling evidence for the involvement of the endocannabinoid system (ECS) in modulating synovial inflammation and nociception within the complex pathogenesis of kOA. While the current data provides fundamental descriptive observations of expression levels and correlations in a well-powered human cohort, it offers valuable foundational insights into patient potential responsiveness to targeted CB2R-specific intervention. This is particularly crucial given the limitations of current non-operative therapies, such as corticosteroids and NSAIDs, which offer only transient pain relief and fail to modify the underlying chronic inflammation and fibrosis that drive disease advancement. Notably, some NSAIDs may even carry risks of chondrotoxicity or accelerating disease progression [6]. The identification of specific CB2R-positive cells and deeper mechanistic insights is the subject of ongoing investigation.

The data reveal a significant inverse relationship between synovial CB2R expression, patient-reported pain (as measured by KOOS), and histological synovitis. This observation suggests that targeting CB2R could potentially limit inflammatory joint damage by modulating the activity of synovial fibroblasts and macrophages while concurrently altering nociceptive signaling [13,36]. It further implies that individuals experiencing lower pain levels may possess a more effectively regulated ECS, characterized by reduced chronic activation and, consequently, less CB2R desensitization and downregulation. This preserved receptor availability could explain the higher CB2R expression observed in the low-pain cohort.

A significant challenge in managing kOA is the frequent decoupling between subjective patient-reported symptoms and objective measures of disease severity, such as radiographic findings or range of motion [8,37]. This discrepancy, where imaging often reveals asymptomatic kOA, suggests that factors beyond gross structural damage, such as the neuroinflammatory state of the synovium, are critical [9]. A subset of patients develops hyperalgesic kOA, marked by spontaneous pain and heightened sensitivity to stimuli, often necessitating opioid use and significantly diminishing quality of life [38]. This hyperalgesia may stem from peripheral and central sensitization mechanisms, further complicating the pain phenotype. Pathological changes within the synovium, particularly synovitis-induced neuroinflammation and hyperalgesia, appear crucial in driving symptom severity and disease progression. The highly innervated synovium undergoes inflammatory and fibrotic remodeling, altering microvascularity and nociceptive signaling, likely contributing substantially to kOA arthralgia [9]. This points toward a neurogenic inflammatory basis for kOA pain, with TRPV1 upregulation identified as a key factor in synovial hyperalgesia [13].

The results demonstrate significantly increased TRPV1 expression in synovial tissues from patients reporting high pain levels. TRPV1 is known to be activated by a range of noxious stimuli, including heat, inflammatory cytokines (such as those found in kOA), and the acidic pH characteristic of inflamed tissues [13,36,39]. The co-localization of TRPV1 with the neuronal marker PGP9.5 suggests that TRPV1 is present on both nociceptive nerve fibers in the subintima and potentially on hyperplastic synoviocytes in the intima, consistent with its expression on non-neuronal synovial cells [20]. This finding suggests a strong association between local synovial pathology and neurogenic pain signaling, with TRPV1 upregulation appearing to be a key molecular event, involving the release of neuropeptides such as CGRP, which promote neurogenic inflammation and hyperexcitability [9]. Our data suggest that, in combination with local CGRP levels of the SF, the distribution of TRPV1 in synovial tissues can help indicate pain severity. Patients exhibiting pain hypersensitization often display hyperexcitable responses to sensory input, complicating pain management. Therefore, quantifying synovial TRPV1 distribution could help identify samples from patients with pain hypersensitization who might benefit most from therapies targeting this pathway.

The chronic pro-inflammatory milieu, rich in cytokines (e.g., IL-1β, transforming growth factor-beta (TGF-β), and TNF-α) released by activated macrophages and infiltrating lymphocytes within an acidic microenvironment, may drive the elevated TRPV1 expression observed in high-pain patients, which can alter receptor turnover and sensitivity [40]. This is supported by findings that link such inflammatory mediators to increased nerve fiber density and hyperalgesia [36]. Chronic TRPV1 activation by these inflammatory and potentially mechanical stimuli likely promotes local receptor upregulation and the development of hypersensitive pain responses. This chronically active TRPV1 pathway will also lead to increased 2-AG levels in the local environment, known to be synthesized in response to TRPV1 activation [24].

The observation that TRPV1 expression was elevated in the high-pain group without a concomitant significant increase in overall synovial nerve fiber density (PGP9.5) is particularly noteworthy. While inflammation can lead to a decrease in nerve fiber density [11], this suggests that the increased TRPV1 signal is not merely a reflection of greater innervation, but rather an increase in the TRPV1 channel on existing nerve fibers and/or non-neuronal synoviocytes. This points to a qualitative change in the nociceptive apparatus within the synovium of high-pain patients.

Conversely, the finding that synovial CB2R distribution is lower in patients presenting with highly severe synovitis and pain aligns with what results from chronic CB2R activation. CB2R agonism on human osteoarthritic fibroblast-like synoviocytes has been shown to suppress M1 macrophage polarization and subsequent inflammation [36]. However, persistent ECS activation in chronic inflammatory states, such as kOA, can lead to a desensitized (unresponsive) and downregulated CB2R [31], potentially diminishing its protective anti-inflammatory capacity. This phenomenon explains the observation of lower CB2R expression in the high-pain/high-synovitis group. It also provides a plausible explanation for the elevated 2-AG levels in the SF of high-pain patients. While 2-AG is typically undetectable in healthy SF, it is markedly elevated in patients with kOA [41]. As shown in Figure 3B, CB2R is typically coupled to an inhibitory G protein, Gαi/o, an "off switch" for inflammation​. When activated at normal physiological levels, this leads to the inhibition of adenylate cyclase, resulting in reduced cyclic adenosine monophosphate (cAMP) and the suppression of pro-inflammatory cytokine expression. This suppression of cytokine expression is achieved by inhibiting transcription factors, such as NFκB, a master regulator of pro-inflammatory cytokine production [42]. This is in contrast to what may take place under states of sustained inflammation, where overactivation of CB2R is observed and a possible paradoxical shift to coupling with a stimulatory G protein, Gαs​, can occur as an "on switch" of inflammation, which has primarily been demonstrated in leukocytes [43]. As shown in Figure 3C, if operative in synoviocytes, progression down this stimulatory pathway could lead to elevated cAMP levels, thereby activating the NFκB pathway and resulting in increased pro-inflammatory cytokine production and inflammation [44].

Furthermore, chronic inflammation, which causes the production of noxious stimuli, can result in the constant activation of TRPV1, driving increased 2-AG production in turn [24]. This increase in 2-AG in the local environment can overwhelm the ECS and CB2R, leading to CB2R downregulation and agonist accumulation, thereby diminishing its therapeutic effects (Figure 3C). Moreover, endocannabinoids like 2-AG can be metabolized via Cox-dependent pathways to pro-inflammatory prostaglandins, which can drive downstream mediators such as IL-6, TNF-α, and IL-1β linked to synovitis and hyperalgesia [45]. Elevated 2-AG in cerebrospinal fluid (CSF) and SF has also been associated with increased acute postoperative pain following TKA, further suggesting a pro-inflammatory role for accumulated 2-AG in settings of chronic inflammation and CB2R downregulation [46]. These findings strongly support the involvement of CB2R and the ECS in regulating TRPV1-mediated neuroinflammatory pain.

Preclinical studies demonstrate that endocannabinoids (like AEA, which also binds TRPV1 with affinity similar to capsaicin) and selective exogenous CB2R agonists (like JWH133) act as partial agonists at TRPV1 [47]. These interactions highlight TRPV1 as an "ionotropic cannabinoid receptor" [47]. They may induce a small inward calcium current, insufficient to trigger nociceptor firing but adequate to desensitize TRPV1 via calcium-dependent mechanisms such as calcineurin activation or by affecting TRPV1 trafficking [48]. The observation that CB2R-mediated analgesia is attenuated in TRPV1 knockout mice further substantiates that functional crosstalk between CB2R and TRPV1 contributes significantly to the analgesic mechanism via interplay between the ECS and EVS [48]. This implies that while targeting CB2R alone may offer baseline analgesia, therapeutic strategies leveraging the CB2R-TRPV1 crosstalk, potentially through CB2R-triggered endocannabinoid release that then modulates TRPV1 or direct receptor interactions, could achieve a greater analgesic effect.

The results advocate for investigating synthetic CB2R agonists, capable of engaging this crosstalk and desensitizing TRPV1, as potential disease-modifying analgesics for kOA. This aligns with the understanding of TRPV1's "dual role" as a "pain switch," capable of both sensitization and desensitization [49]. The concept of "functional selectivity", where a single therapeutic agent can be engineered to activate only a specific, desired signaling pathway, in CB2R signaling emphasizes that different agonists can produce distinct cellular responses by activating differing repertoires of signaling pathways [50]. Administering such agonists earlier in the disease course might help maintain ECS homeostasis, preventing the cycle of overactivation, desensitization, and downregulation observed in chronic inflammation. Preclinical evidence also supports the ability of peripherally administered cannabinoid agonists to attenuate hyperalgesia [51]. The synthetic CB2R agonist JWH133, for instance, reduces inflammatory mediators and synovitis in animal models [36]. JWH133 exhibits antioxidant, anti-fibrotic [52], and anti-inflammatory properties [36] without the hepatotoxicity reported for CBD [26]. This suggests that selective CB2R agonists could offer superior pain relief and potentially slow disease progression through multifaceted mechanisms, including inhibiting fibroblast production of IL-6/IL-8 and modulation of TGF-β1 [53]. Future research should also evaluate the ability of select allosteric agonists, alone or in combination with orthosteric activators, to restore adequate CB2R distribution or functionality, including the use of novel bitopic ligands, advanced therapeutic molecules designed to bind to a receptor in two different places at once for a more specific and potent effect [54]. If the CB2R can be favorably modulated using synthetic agonists, this may decrease the accumulation of endogenous agonists like 2-AG, which will limit ECS dysregulation and further inflammation.

This study has several limitations. First, our cross-sectional design, while providing valuable correlational evidence, cannot establish a direct causal relationship between the observed receptor expression levels and pain severity. The findings demonstrate a strong association between a dysregulated CB2R/TRPV1 axis and pain, but they do not prove that altering receptor expression will directly cause changes in pain perception. Second, the lack of a true non-OA control group limits our ability to make conclusions about baseline CB2R and TRPV1 expression levels in healthy synovium. However, our robust stratification of a clinically homogeneous population into high- and low-pain groups allowed us to identify critical molecular differences that exist even among patients with end-stage disease, which may be more relevant for predicting therapeutic responsiveness. Finally, the logistical challenges of intraoperative sample collection led to natural variability in sample sizes across different assays. Although we maximized our use of all available samples, as shown in Table 2, this variability suggests that some conclusions, especially regarding CGRP and 2-AG levels, are based on a smaller subset of the overall patient group and need to be confirmed with additional studies.

**Table 2:** Sample size (n) limitations per assay PS: Picrosirius, Q-IF: quantitative immunofluorescence, ELISA: enzyme-linked immunosorbent assay, CGRP: calcitonin gene-related peptide, LC-MS/MS: liquid chromatography-tandem mass spectrometry, 2-AG: 2-arachidonoylglycerol, AEA: anandamide

Assay	Total (number)	Low pain (number)	High pain (number)
Histology (PS)	73	32	41
Q-IF	73	32	41
ELISA (CGRP)	44	23	21
LC-MS/MS (2-AG, AEA)	44	23	21

Future studies are warranted to validate these findings and to incorporate functional assays that can directly measure receptor activity.

## Conclusions

This study provides compelling evidence for a dysregulated TRPV1/CB2R axis in painful knee osteoarthritis synovitis, characterized by reduced synovial CB2R expression and elevated TRPV1 expression in patients with higher pain and synovitis. The increased levels of 2-arachidonoylglycerol (2-AG) in synovial fluid further underscore a complex interplay within the endocannabinoid system (ECS) that may contribute to chronic inflammation and pain. These findings suggest that the protective functions of CB2R are compromised in severe kOA, potentially due to chronic agonist-induced desensitization and a shift toward pro-inflammatory signaling pathways. The intricate crosstalk between CB2R and TRPV1 offers a promising avenue for therapeutic intervention. Selective CB2R agonists, which can modulate TRPV1 activity without the adverse effects associated with direct TRPV1 targeting or non-specific cannabinoid activation, represent a novel strategy for managing kOA pain. By restoring CB2R function and leveraging its interaction with TRPV1, these agents could provide both analgesic and disease-modifying benefits, addressing the critical unmet need for treatments that go beyond symptomatic relief. Future research should focus on elucidating the precise cellular mechanisms underlying CB2R dysregulation and exploring the efficacy of targeted CB2R modulators to develop personalized and effective therapies for this debilitating disease.
